# The Impact of Language Switching Frequency on Attentional and Executive Functioning in Proficient Bilingual Adults

**DOI:** 10.5334/pb.392

**Published:** 2018-05-16

**Authors:** Cristina Barbu, Sarah Orban, Sophie Gillet, Martine Poncelet

**Affiliations:** 1 Psychology and Neuroscience of Cognition Research Unit, University of Liège, Liège, BE

**Keywords:** language-switching frequency, response inhibition, cognitive flexibility, alerting

## Abstract

Bilingual advantages in executive functions are well documented (see Bialystok, 2009; Dong & Li, 2015, for a review), but the specific aspects of bilingualism that underlie these advantages are unclear. The few studies conducted up until now on this subject (e.g., Hartanto & Yang, 2016; Prior & Gollan, 2011; Verreyt, Woumans, Vandelanotte, Szmalec, & Duyck, 2016) have suggested that the frequency of language switching may partially mediate this advantage. We further investigate the impact of oral language-switching frequency on the development of alerting, response inhibition and cognitive flexibility skills in proficient bilinguals. Two groups of proficient bilingual adults (21 low-frequency language switchers and 21 high-frequency language switchers), matched for age, gender, second-language proficiency and socio-cultural status, participated in the study. Tasks assessing alerting, response inhibition and cognitive flexibility were administered. Our results revealed that high-frequency language switchers responded more quickly in the task assessing cognitive flexibility. No group effect was found on the tasks assessing alerting and response inhibition. These results suggest that language-switching frequency is likely an underlying factor in the enhanced cognitive flexibility of proficient bilinguals.

## Introduction

Executive functioning refers to a set of higher-order control processes designed to ensure the adaptation of an individual to different environmental demands (Collette, 2004). According to the model proposed by Miyake et al. (2000), executive functioning consists of three core processes: inhibition-related functions, information updating, monitoring (or working memory), and mental-set shifting. Executive functioning has been shown to be positively influenced by bilingualism (Bialystok, 2009). Bilingual executive advantages have been observed in children (e.g., Bialystok & Barac, 2012; Kalashnikova & Mattock, 2014; Nicolay & Poncelet, 2013; Nicolay & Poncelet, 2015), adults (e.g., Bialystok, Poarch, Luo, & Craik 2014; Costa, Hernandez, Costa-Faidella, & Sebastián-Gallés, 2009; Costa, Hernandez, & Sebastián-Gallés, 2008; Fernandez, Acosta, Douglass, Doshi, & Tartar, 2014; Fernadez, Tartar, Padron, & Acosta, 2013; Ibrahim, Shoshani, Prior, Prior, & Share, 2013; Marzecová, Asanowicz, Kriva, & Wodniecka, 2012; Seçer, 2016) or even older adults (e.g., Bialystok et al., 2014). These advantages, demonstrated by several studies, have been noted in tasks assessing, for instance, the suppression of automatic response tendencies (response inhibition; Fernandez et al., 2013; Fernandez et al., 2014), the ability to ignore irrelevant conflicting information (interference inhibition; Costa et al., 2008; Marzecová et al., 2012), the ability to shift from a mental set to another (set-shifting) within the demands of a particular context or to switch attention from one aspect to another of a stimulus (cognitive flexibility; Ibrahim et al., 2013; Liu, Fan, Rossi, Yao, & Chen, 2015; Nicolay & Poncelet, 2013, 2015; Seçer, 2016), and the ability to monitor and to use (or to delete) working memory information (updating; e.g., Dong & Li, 2015). All these skills cover the range of executive sub-components (inhibition, cognitive shifting or cognitive flexibility, and updating) described by the model of Miyake (Miyake et al., 2000). Bilingual advantages have also been showed in tasks assessing alerting (Costa et al., 2009; Costa et al., 2008; Marzecová et al., 2012; Nicolay & Poncelet, 2013, 2015). Alerting is the ability to react rapidly and adequately to environmental changes (Leclercq & Zimmerann, 2000). It is a basic function which is likely required in all executive tasks.

Previous studies have attributed these benefits to bilingualism as a global factor. According to Green (1998), bilingualism requires the control of the two simultaneous activated languages and more specifically inhibiting lexico-semantic competitors of the non-intended language. This increased recruitment and training of inhibitory skills appears to improve executive functioning. Other authors (e.g., Costa, Pannunzi, Deco, & Pickering, 2016) have attributed these advantages to the transfer of structures of the native to the non-native language during language acquisition.

The bilingual advantages in executive functioning have not been constantly observed though and are therefore controversial (e.g., Paap & Greenberg, 2013; Paap, Johnson, & Sawi, 2014). This lack of consistency has been attributed to different un-controlled non-linguistic factors (i.e., socioeconomic status (Hackman, Gallop, & Evans, 2015), video game or music practice (Boot, Kramer, Simons, Fabiani, & Gratton, 2008) or linguistic factors including second language (L2) proficiency (e.g., Fernandez et al., 2013) or language-switching frequency (e.g., Hartanto & Yang, 2016; Prior & Gollan, 2011; Soveri, Rodriguez-Fornells, & Laine, 2011; Verreyt et al., 2016). In terms of language switching, a few recent studies (e.g., Hartanto & Yang, 2016; Prior & Gollan, 2011; Verreyt & al., 2016) have indeed suggested that switching frequently between languages or language-switching frequency might improve the development of executive functioning. Language-switching occurs either in two-language contexts in which bilingual individuals change languages mid-utterance when speaking with another bilingual, or in one-language contexts where bilinguals speak one language, for instance L1 at a given time with an L1 monolingual and then L2 at another time with an L2 monolingual.

Despite the large number of studies demonstrating a bilingual advantage in tasks assessing response inhibition and interference inhibition skills, only Verreyt et al. (2016) attempted to determine the influence of language-switching frequency on the development of inhibition skills (interference inhibition) in bilinguals. In addition, no study to date has tried to specifically assess the effect of language-switching frequency on response inhibition skills in bilinguals. Verreyt et al. (2016) compared proficient (balanced) Dutch-French bilinguals who switched frequently between languages with proficient (balanced) Dutch-French bilinguals who seldom switched between languages and non-switching less proficient (imbalanced) Dutch-French bilinguals in a task assessing interference inhibition skills (Attentional Network Task: ANT; Fan, McCandliss, Sommer, Raz, & Posner, 2002). In this task, participants were presented with five arrows (pointing leftwards or rightwards) on a computer screen and asked to indicate the direction of the target arrow occurring in the middle. A conflict effect was calculated by subtracting the mean response speed from the condition in which all of the arrows were pointing in the same direction (congruent condition) from the one in which the central arrow was pointing in the opposite direction (incongruent condition). Verreyt et al. (2016) found that proficient (balanced) Dutch-French bilinguals who switched frequently between languages exhibited a faster response speed compared with both proficient (balanced) Dutch-French bilinguals who seldom switched between languages and non-switching less proficient (imbalanced) Dutch-French bilinguals. The results of this study also failed to find any group difference between non-switching proficient (balanced) bilinguals and non-switching less proficient (imbalanced) bilinguals in this task. The authors argued that language-switching frequency improves the development of interference resolution skills (or interference inhibition) in bilinguals. This advantage has been seen as reflecting bilinguals’ ability to inhibit second-language intrusions from the non-intended language when switching between languages.

Moreover, despite several studies demonstrating that bilingualism enhances cognitive flexibility skills (Ibrahim et al., 2013; Prior & Gollan, 2011; Liu et al., 2015; Nicolay & Poncelet, 2013, 2015; Hartanto & Yang, 2016; Seçer, 2016), only two studies to date have assessed the impact of language-switching frequency on the development of switching skills involving cognitive flexibility in bilinguals (Prior & Gollan, 2011; Hartanto & Yang, 2016). Prior and Gollan (2011) showed that proficient Spanish-English bilinguals who switched frequently between languages exhibited enhanced task-shifting skills (faster response speed) compared with proficient Mandarin-English bilinguals who switched less frequently between languages and English monolinguals. Hartanto and Yang (2016) observed similar findings when comparing the performance of two groups of proficient bilinguals (i.e., high- and low-frequency language switchers) in a task assessing switching skills. Both studies used a similar task to assess switching skills (e.g., Paap & Greenberg, 2013). In both tasks, participants were asked to switch from one type of trial to the other (color or shape). These tasks both required two types of costs: switching and mixing costs. Switching costs refer to response speed delays on switch trials. Mixing costs, on the other hand, refers to task-repeat trials in mixed-task blocks compared with single-task blocks. In both studies, the results revealed no group differences in correct responses or mixing costs. For the switching costs, however, high-frequency language switchers outperformed low-frequency language switchers. Both Prior and Gollan (2011) and Hartanto and Yang (2016) argued that reduced switching costs in this task reflect enhanced shifting skills in individuals who switch frequently between languages.

Finally, despite several studies assessing the impact of bilingualism on alerting skills (e.g., Costa et al., 2008; Marzecová et al., 2012; Nicolay & Poncelet, 2013, 2015), no study to date has sought to determine whether language-switching frequency influences the development of alerting skills in proficient bilinguals.

As noted above, several studies have revealed that bilingualism, as a general factor, enhances response inhibition, cognitive flexibility and alerting in proficient bilinguals. These advantages might, however, not be due to bilingualism as such but rather to specific linguistic factors related to bilingualism such as language-switching frequency. However, no study or very few studies to date have investigated the effect of language-switching frequency on the development of these skills in proficient bilinguals. The aim of this study was to investigate the effect of oral language-switching frequency on the development of response inhibition, cognitive flexibility and alerting in proficient bilinguals.

We hypothesize that switching orally between languages recruits response inhibition, cognitive flexibility and alerting. These skills are necessary to prevent intrusions from the non-intended language (response inhibition), to switch from one mental set to another (cognitive flexibility) and to maintain a constant preparation state (alerting) (e.g., Nicolay & Poncelet, 2013; 2015; Prior & Gollan, 2011; Prior & MacWhinney, 2010; Verreyt et al., 2016) when switching between languages. In this context, we should expect to observe an advantage in proficient bilinguals (who switch frequently between languages) compared with bilinguals (who rarely switch between languages) in tasks assessing these skills.

## Method

### Participants

A total of 51 bilingual adults (41 women and 10 men) were recruited from different regions of Belgium. All participants had a high level of proficiency in L2, as estimated by self-rated L2 skills in speaking, reading, writing and speech comprehension, and by an assessment of receptive L2 vocabulary skills via an adaptation of the BPVT (British Picture Vocabulary Test: Dunn, Dunn, Whetton, & Pintilie, 1982) corresponding to the participants’ L2. All of the participants spoke French as either their L1 or L2. The participants also spoke a range of other languages (see description below). None of the participants had a specific professional background that required strong language-switching skills (e.g., simultaneous translation). They had no specific language or neurological impairment, brain injury, attentional, auditory or visual deficit at the time of testing. From the total population of 51 bilinguals, two groups with very contrasting language-switching frequencies were selected: one group of 21 proficient bilinguals switching orally between language a minimum of 20 times per day (i.e., HFLS; mean language-switching frequency: 54.15 ± 34.96) and one group of 21 proficient bilinguals switching orally a maximum of 6 times per day (i.e., LFLS; mean language-switching frequency: 2.91 ± 2.26). The remaining 9 participants that switched orally between 7 and 19 times per day (mean language-switching frequency: 11.11 ± 3.37) were discarded from the study. The results obtained by HFLS and LFLS were the only ones included in the analysis.

The HFLS group was composed of 16 women and 5 men between the ages of 18 and 43 years (M = 26.00; SD = 6.11). They had a variety of first languages, including German (15), French (3), Romanian (1), English (1) and Dutch (1). Their most proficient second language was French (18), English (1), and German (2). The L1–L2 pairs of participants in this group combined different language families, including Germanic-Romance (17), Romance-Germanic (3) and Romance-Romance (1). The LFLS group was composed of 17 women and 4 men between the ages of 18 and 42 years (M = 25.52; SD = 6.65). They were first-language speakers of a variety of languages, including German (5), French (7), Romanian (2), Portuguese (2), Italian (2), Russian (1), Spanish (1) and Kirundi (1). Their most proficient second languages included French (14), Dutch (2), English (3) and German (2). The L1–L2 language pairs of the participants in this group combined a number of language families, including Romance-Germanic (7), Germanic-Romance (5), Romance-Romance (7), Slavic-Romance (1) and Bantu-Romance (1). The two language switching groups were matched in terms of age, gender, socio-cultural status, video game and music practice, self-rated L2 proficiency, and L2 receptive vocabulary skills (detailed results are presented in Table 1). Low-frequency switchers, however, had more L1–L2 pairs within the same language family than high-switching bilinguals (7 vs. 1).

**Table 1:** Descriptive statistics, mean comparisons between high- and low-switching bilinguals by using inferential and Bayesian statistics on age, socio-cultural status, video game practice, second-language receptive vocabulary, and self-rated second language proficiency.

	High-frequency switchers N = 21	Low-frequency switchers N = 21	Inferential statistics		Bayesian statistics	

	Mean (SD)	Mean (SD)	t	p	BF_10_	BF_10_ (error %)

Age (years)	26.00 (6.11)	25.52 (6.65)	–0.24	0.81	0.310	1.400e–4
Academic background (years of education)	15.71 (1.61)	15.38 (2.76)	–0.47	0.63	0.332	1.409e–4
Video game practice (h/week)	0.35 (0.85)	0.04 (0.21)	–1.61	0.11	0.843	9.040e–5
Receptive vocabulary level-standard scores (z scores)	0.68 (0.75)	0.88 (1.07)	0.68	0.49	0.365	1.443e–4
Global score for self-rated second language proficiency (max = 24)	21.83 (1.79)	20.90 (1.78)	–1.67	0.10	0.920	8.154e–5

df = 40; h = hours; BF_10_ = Bayes factor for the alternative hypothesis vs. the null hypothesis. Bayes factor is undefined – one or both levels of the dependent contain all the same value (zero variance).

### Materials and procedures

We distributed a general questionnaire that collected information concerning the participant’s age, medical history (specific language impairment, brain injury, and auditory deficits), socio-cultural status (total number of years of study completed since first grade), video-game practice (total number of hours per week), a range of linguistic variables such as language exposure during lifetime in school, age at onset of L2 exposure, self-rated level of L2 proficiency and daily oral language switching frequencies (see the detailed description below).

### Assessment of language skills and language practice

We measured second-language proficiency by asking the participants to rate their proficiency in speaking, reading, writing and speech comprehension on a 6-point Likert scale (from 1 = very low to 6 = very high). A global score was calculated to obtain a total estimate of second-language proficiency. Furthermore, different versions of the BPVT (British Picture Vocabulary Test: Dunn et al., 1982) adapted to German (Peabody Picture Vocabulary Test: Dunn & Dunn, 1997), French (Echelle de Vocabulaire en Images Peabody: Dunn, Thériault-Whalen, & Dunn, 1993) and Dutch (Peabody Picture Vocabulary Test: Dunn & Dunn, 2005) were also applied to measure L2 proficiency. Participants were assessed with the measure for their particular L2. In all four of the versions used, for each item, the participants were shown four images on a computer screen, the experimenter spoke a word and the participants were asked to say the number of the corresponding image. The items were ordered by increasing level of difficulty. The four versions differed in the total number of items included. The testing procedure was applied according to instructions of the different test versions used. Raw scores were converted into standard scores (z scores) for analysis. Standard scores (z scores) were also used to ensure the comparability of the different test versions. Only the L2 proficiency level of participants was measured given that their L1 corresponded to their mother tongue, a language assumed to be already highly mastered. We measured language-switching frequency by asking participants to estimate the number of times they switched orally between languages within a day. More precisely, we demanded participants to estimate and to sum up the total number of times they switched orally between languages over the course of a week. We then divided that number by seven to establish the average number of times participants switched between languages within the course of a day.

### Executive measures

The tasks used to assess response inhibition, cognitive flexibility and alerting derived from the Test of Attentional Performance (TAP) battery (Zimmermann & Fimm, 2009), a computerized standardized test battery that measures various aspects of attention. Each of the tasks is presented below.

Alerting skills were assessed using the Alertness subtest from the TAP battery. In this task, participants were asked to respond by pressing a key as quickly as possible when a visual stimulus (an “×” sign) appeared at the center of the screen. The task comprised 20 trials from which the first two were dummies. We recorded the reaction times and total errors of each participant. Errors in this task were considered as very long responses (=RT superior to mean + 2.35 × standard deviation).

We measured response inhibition using the Go/Nogo subtest from the TAP battery. Participants were asked to press a key response as quickly as possible when an “×” sign (target) appeared at the center of the screen, and to withhold their response when a “+” sign (distractor) appeared instead. This task includes 40 trials (20 targets and 20 distractors). Each stimulus is presented for maximum 200 milliseconds. We recorded the reaction times and total errors of each participant.

We measured cognitive flexibility using the Flexibility subtest from the TAP battery. In this task, participants were asked to alternate between two types of stimuli (letters and numbers), one of which appeared randomly on either the right or the left side of the computer screen. There were two response keys, one corresponding to the right side of the screen and the other to the left side of the screen. The participants’ task alternated from trial to trial: in one trial, they were asked to press the response key corresponding to the side the letter was on, in the next trial they were asked to press the response key corresponding to the side of the number, on the third the letter again, and so on in alternation. Acoustic feedback was given for errors. The task comprised 100 trials and lasted for approximately 3.5 minutes. Reaction times and total number of errors were recorded.

### General procedure

The various tasks were all administered in French in a single individual testing session, which lasted between 1.5 and 2.0 hours.

The different tasks were administered in a fixed order. First, participants performed the executive tasks from the TAP battery, followed by the L2 receptive vocabulary task. Testing continued with the administration of the questionnaire. In all cases, the participants were seated at a comfortable distance from the computer screen.

**Statistical analyses**. We compared the performance of participants in the experimental measures using *t*-tests (independent sample *t*-tests) and chi-squared tests. In light of the critiques of an indirect approach to inferential statistics regarding biases with respect to the null hypothesis, statistical power and p-values (Wagenmakers, 2007; Wagenmakers et al., 2015) we also used Bayesian *t*-tests and Bayesian Pearson correlations (Love et al., 2015; https://jasp-stats.org/). This approach enables an unbiased estimation of the evidence for a model that includes a group effect or a between measure correlation as compared to a null model in which there is no group effect or no between measure correlation. In Bayesian statistics, there is no significance threshold. However, the larger the Bayes factor associated with a given model, the stronger the evidence in favor of this model compared with the null model. A Bayes factor above 3 is considered to be moderate evidence, a Bayes factor above 10 is considered to be strong evidence and a Bayes factor above 30 is considered to be very strong evidence (Lee & Wagenmakers, 2014).

## Results

### Preliminary measures

A series of *t*-tests revealed no significant differences between the groups in terms of age, socio-cultural level, video-game practice, receptive vocabulary skills and self-rated second-language proficiency. Two additional chi-square tests were also used to compare the two language groups in terms of gender and number of subjects involved in music training. The results revealed no group difference in terms of gender, χ^2^ (1) = 0.14, *p* = 0.70 or number of subjects involved in music training, χ^2^ (1) = 0.46, *p* = 0.49. Bayesian *t*-tests were also conducted on control measures of age, academic background, receptive vocabulary levels, video game practice, and self-rated levels of L2 proficiency. The results revealed that the Bayes factors of the alternative model (including a group effect) were only 0.31 for age, 0.33 for academic background, 0.36 for receptive vocabulary levels, 0.84 for video game practice and 0.92 for self-rated levels of L2 proficiency. These results provide no evidence for a group difference on these control measures (detailed results are presented in Table 1).

### Executive measures

A *t*-test (Love et al., 2015; https://jasp-stats.org/) carried out on the accuracy data revealed no significant group difference in the alerting task (*p* = 0.39; range for low-switching bilinguals: 0–2 errors per 18 items; range for high-switching bilinguals: 0–1 errors per 18 items), in the response inhibition task (*p* = 1; range for low-switching bilinguals: 0–4 errors per 20 items; range for high-switching bilinguals: 0–3 errors per 20 items) or in the cognitive flexibility task (*p* = 0.44; range for low-switching bilinguals: 0–12 errors per 100 items; range for high-switching bilinguals: 0–16 errors per 100 items). Moreover, no speed-accuracy trade-off was observed for this former task based on a correlation analysis conducted between reaction time and error rates (r = 0.02; *p* = 0.87).

The *t*-tests that we used to compare high- and low-switching bilinguals on reaction time for measures of response inhibition, cognitive flexibility and alerting revealed a significant group difference only in cognitive flexibility, t(40) = 2.20 (*p* = 0.03), with high-switching proficient bilinguals performing faster than low-switching proficient bilinguals. Concerning the cognitive flexibility task, the results of the Bayesian analysis for response speed suggested that the alternative model that included a group effect was over three times more likely than the null model with no group effect (BF_10_ = 3.875). For error rates, however the Bayes factor was only of 0.57. Our results also revealed that for the alerting task the Bayes factor of the alternative model was only 0.30 for response speed and 0.40 for errors. Concerning the response inhibition task, the Bayesian analysis revealed that the Bayes factor was only of 0.34 for response speed and of 0.30 for errors rates. Additional analyses for the cognitive flexibility task revealed that compared with the alternative model, the Bayes factor of the null model including no group effect was only 0.25 for response speed and of 1.72 for errors. In contrast, for the alerting task, the null model was approximately three times more likely for response speed and two times more likely for errors. For the response inhibition task, the Bayesian analysis revealed that the null model was two times more likely for response speed and three times more likely for error rates. Detailed results are presented in Table 2.

**Table 2:** Descriptive statistics, mean comparisons by using inferential and Bayesian statistics in measures of alerting, response inhibition and cognitive flexibility (reaction times in milliseconds, and errors).

	High-frequencyswitchers N = 21	Low-frequency switchers N = 21	Inferential statistics			Bayesian statistics			

	Mean (SD)	Mean (SD)	t	p	Effect size (Cohen’s d)	BF_10_	BF_10_ (error %)	BF_01_	BF_01_ (error %)

Alerting RT (msec)	238.80 (43.17)	239.52 (34.07)	0.06	0.95	0.01	0.303	~1.400e–4	3.296	~1.400e–4
Alerting Errors (max = 18)	0.71 (0.46)	0.57 (0.59)	–0.86	0.39	–0.26	0.409	~1.499e–4	2.445	~1.499e–4
Response inhibition RT (msec)	393.14 (67.02)	404.61 (68.03)	0.55	0.58	0.17	0.342	~1.417e–4	2.922	~1.417e–4
Response inhibition Errors (max = 20)	0.90 (0.99)	0.90 (1.22)	0	1	0	0.303	~1.400e–4	3.300	~1.400e–4
Cognitive flexibility RT (msec)	551.19 (120.32)	645.66 (155.47)	2.20	0.03	0.68	3.875	~6.004e–5	0.258	~6.004e–5
Cognitive flexibility Errors (max = 100)	2.33 (2.28)	2.85 (2.10)	0.77	0.44	0.23	0.579	~1.469e–4	1.728	~1.469e–4

df = 40; RT = reaction time in milliseconds (msec). BF_10_ = Bayes factor for the alternative hypothesis vs. the null hypothesis. BF_01_ = Bayes factor of the null hypothesis vs. the alternative hypothesis.

Given the significant group difference observed in the cognitive flexibility task (*p* = 0.03), an additional Bayesian correlation analysis was conducted between oral language-switching frequencies reported by the total initial population of 51 bilinguals and response speed in the cognitive flexibility task in order to further explore the relationship between language-switching frequency and improved cognitive flexibility skills in proficient bilinguals. Results revealed that the model containing a between measure correlation was three times more likely as compared to the model containing no between measure correlation (BF_10_ = 3.93; r = –0.35). These results provide moderate evidence that language-switching frequencies are correlated with response speed in the cognitive flexibility task.

All the above mentioned findings are consistent with the results obtained with traditional statistics, and they appear to suggest that oral language switching frequency has a positive impact on the development of cognitive flexibility in proficient bilinguals. In contrast, they offer no significant evidence for a positive effect of oral language switching frequency on the development of response inhibition and alerting in proficient bilinguals.

## Discussion

The aim of this study was to examine the effect of oral language switching frequency on the performance of proficient bilinguals in tasks assessing response inhibition, cognitive flexibility and alerting skills.

We hypothesized that response inhibition, cognitive flexibility and alerting skills are recruited and accordingly trained by oral language switching. Response inhibition would be required in order to avoid intrusions of the non-desired language. Cognitive flexibility would also be needed to shift between mental sets. Finally, alerting would be necessary in order to maintain a constant alerting state when processing L2 (e.g., Nicolay & Poncelet, 2013, 2015; Prior & Gollan, 2011; Prior & MacWhinney, 2010; Verreyt et al., 2016). In this context, proficient bilinguals who switch languages often would outperform proficient bilinguals who rarely do so in tasks assessing response inhibition, cognitive flexibility and alerting.

As predicted, our results revealed a significant group advantage in terms of cognitive flexibility skills. More precisely, HFLS responded more quickly than LFLS on the task assessing cognitive flexibility. However, no significant group difference was observed on the response inhibition and alerting tasks. The faster response speed of HFLS in the cognitive flexibility task cannot be accounted for by differences in age, gender, video-game practice, socio-cultural status or L2 proficiency since there were no differences between the groups in any of these control measures. The group difference on the cognitive flexibility task may also not be accounted for differences on basic attentional processes, which underlie cognitive flexibility such as alerting given that no group differences on the alerting task were observed.

The advantage observed in HFLS in the cognitive flexibility task could be explained by the fact that the task used to assess this skill relies on task-switching skills given that participants are required to distinguish the stimuli on the basis of membership in a certain abstract category (in this case, either letters or numbers) and to shift between task sets on different (intermingled) items. This task involves processes similar to language switching in which constant abstract language categorization and language-set shifting are necessary. These results support previous findings (e.g., Prior & Gollan, 2011) showing that the performance of bilinguals who frequently switch languages in tasks assessing switching skills is faster than that of bilinguals who rarely switch.

Unlike the cognitive flexibility task used in this study, which relies on switching, the response inhibition and alerting tasks did not require switching: during these tasks participants were mainly asked to inhibit an inadequate stimulus appearing on the screen (response inhibition) or to respond as fast as possible when a simple stimulus (an “×” sign) was present on the screen (alerting).

In order to clearly establish if oral language switching frequency is the underlying factor for the enhanced cognitive flexibility skills in HFLS, forthcoming studies should also investigate the effect of language switching frequency on cognitive flexibility skills by comparing HFLS and LFLS with their monolingual peers. Prior and Gollan (2011) have shown that frequently switching proficient bilinguals exhibit a faster performance (faster response speed) compared with proficient bilinguals who switched infrequently and monolinguals in a task requiring task-shifting skills. No significant group difference was, however, observed between low-frequency switching bilinguals and monolinguals in this task. These findings suggest that oral language switching frequency is a specific factor responsible for the advantage in task-shifting or cognitive flexibility skills in proficient bilinguals.

In conclusion, the results of our study suggest that oral language switching frequency is a specific underlying factor for the enhanced cognitive flexibility in proficient bilinguals. These findings contribute in understanding which specific linguistic factors related to bilingualism is responsible for the bilingual advantage in attentional and executive functioning. They provide evidence that it is not bilingualism as whole, as suggested by previous studies (e.g., Costa et al., 2008; Bialystok, 2009; Bialystok & Barac, 2012; Bialystok et al., 2014), which is responsible for a bilingual advantage in attentional and executive skills but rather language-switching frequency. This linguistic aspect should be assessed by future studies examining the relationships between bilingualism and attentional and executive functioning. Future studies should also further investigate the effect of oral language switching frequency on cognitive flexibility, response inhibition and alerting skills in proficient bilinguals by comparing HFLS and LFLS to their monolingual peers.
